# Molecular Epidemiology of Drug-Resistant Mycobacterium Tuberculosis in Japan

**DOI:** 10.1128/mSphere.00978-20

**Published:** 2021-07-07

**Authors:** Fuminori Mizukoshi, Nobuyuki Kobayashi, Fumiko Kirikae, Ken Ohta, Kazunari Tsuyuguchi, Noritaka Yamada, Yoshikazu Inoue, Masahide Horiba, Noriko Kawata, Akiko Ichinose, Tohru Miyoshi-Akiyama, Reiko Kiritani, Keiji Funatogawa, Teruo Kirikae

**Affiliations:** a Tochigi Prefectural Institute of Public Health and Environmental Science, Utsunomiya, Tochigi, Japan; b Department of Respiratory Medicine, National Hospital Organization (NHO) Tokyo National Hospital, Kiyose, Tokyo, Japan; c Department of Internal Medicine, Fureai Machida Hospital, Machida, Tokyo, Japan; d Department of Microbiology, Faculty of Medicine, Juntendo University, Bunkyo-ku, Tokyo, Japan; e Clinical Research Center, National Hospital Organization Kinki-Chuo Chest Medical Center, Sakai, Osaka, Japan; f Department of Respiratory Medicine, NHO Higashinagoya National Hospital, Nagoya, Aichi, Japan; g Department of Respiratory Medicine, NHO Higashisaitama National Hospital, Hasuda, Saitama, Japan; h Department of Allergy and Respiratory Medicine, NHO Minami-Okayama Medical Center, Tsukubo, Okayama, Japan; i Department of Electrical Engineering and Bioscience, Faculty of Science and Engineering, Waseda University, Shinjuku-ku, Tokyo, Japan; j Department of Infectious Diseases, Research Institute, National Center for Global Health and Medicine, Shinjuku-ku, Tokyo, Japan; k Fukujuji Hospital, Japan Anti-Tuberculosis Association, Kiyose, Tokyo, Japan; Washington University School of Medicine in St. Louis

**Keywords:** drug-resistant *M. tuberculosis*, whole-genome sequencing, foreign born, Japan

## Abstract

Clinical isolates of drug-resistant (isoniazid and/or rifampicin-resistant) Mycobacterium tuberculosis were obtained from 254 patients diagnosed with drug-resistant tuberculosis in Japan from April 2015 to March 2017 in National Hospital Organization hospitals. The 254 patients were approximately 32% of all 795 patients who were diagnosed with culture-confirmed drug-resistant tuberculosis from 2015 to 2016 nationwide in Japan. The whole-genome sequences of all the isolates from the 254 patients and the lineages of these isolates were determined, and phylogenetic trees were constructed based on single nucleotide polymorphism concatemers. Of these patients, 202 (79.5%) were born in Japan and 52 (20.5%) were born elsewhere. Of the 254 drug-resistant isolates, 54 (21.3%) were multidrug resistant, being resistant to both isoniazid and rifampicin. The percentages of multidrug-resistant isolates were significantly higher in foreign-born (38.5% [20/52]) than Japanese-born patients (16.8% [34/202]). Of the 54 multidrug-resistant isolates, nine were extensively drug resistant, which were all obtained from Japanese-born patients. Five extensively drug-resistant isolates were obtained from patients with incipient tuberculosis. A significant number of multidrug-resistant M. tuberculosis strains were isolated from foreign-born patients from Asian countries that have a high tuberculosis burden. Foreign-derived isolates affect the nationwide genetic diversity of drug-resistant M. tuberculosis in Japan. Extensively drug-resistant M. tuberculosis isolates were transmitted among the Japanese population.

**IMPORTANCE** The incidence rate of tuberculosis (TB) in Japan was 11.5 per 100,000 of the population in 2019. Of TB patients in Japan, 61.1% were aged >70 years, and 10.7% were born outside Japan, mostly in Asian countries with a high burden of tuberculosis. Of the tuberculosis patients in the present study, 5.4% and 1.0% showed resistance to isoniazid and rifampicin, respectively, and 0.7% were multidrug resistant. The objective of this study was to clarify the molecular epidemiological properties of drug-resistant tuberculosis in Japan. Molecular epidemiology provides several clues to inform potential measures to control drug-resistant tuberculosis in Japan.

## INTRODUCTION

The World Health Organization (WHO) estimated that in 2019 about 10.0 million people worldwide had developed tuberculosis (TB), among whom approximately 1.45 million died of the disease (1). Most Asian countries, except for Japan, Democratic People’s Republic of Korea, and Singapore, are categorized as countries with a high or relatively high TB burden, which is defined as an incidence rate of ≥100 per 100,000 of the population (1). The prevalence rate of TB in large Japanese cities such as Tokyo is more than twice that in rural areas, which is likely due to larger populations of foreign-born TB-positive immigrants in those cities (2). TB-positive individuals from countries with a high TB burden may introduce globally disseminated isolates of Mycobacterium tuberculosis, not only into large cities such as Tokyo (2), but into rural areas such as Tochigi prefecture (3).

The emergence and spread of multidrug-resistant (MDR) M. tuberculosis have become a global public health threat (1). MDR M. tuberculosis is defined as an isolate resistant to both isoniazid (INH) and rifampicin (RIF), and extensively drug-resistant (XDR) M. tuberculosis is defined as an MDR isolate that is also resistant to fluoroquinolones and any injectable drug (amikacin, kanamycin, or capreomycin) used to treat MDR isolates (1). Approximately half a million new cases of RIF-resistant TB were reported worldwide in 2019, with 78% of those having MDR-TB (1). Globally, 3.3% of newly diagnosed patients and 17.7% of previously treated patients were found to have RIF-resistant or MDR-TB, with approximately 6.0% of RIF-resistant or MDR-TB patients in 2019 having XDR-TB (1). In Japan, 0.7% of TB patients in 2019 were infected with MDR M. tuberculosis, and the percentages of MDR-TB patients did not change in the range between 0.6 and 0.7 from 2012 to 2019 (1, 4).

Foreign-born TB patients from countries with a high TB burden are thought to enhance the risks of M. tuberculosis spread in countries with a low incidence of TB (5). That is, foreign-born TB patients in Japan may be infected with internationally disseminated clones of M. tuberculosis and disseminate these in Japan (2, 3). MDR-TB in European countries with a low incidence of TB is more prevalent among migrants than among the native population (6). In addition, a molecular epidemiological study identified a cluster of MDR M. tuberculosis among patients who were diagnosed with TB in seven European countries, with this strain originating in the Horn of Africa or Sudan (7). Less is known about the population structure of drug-resistant M. tuberculosis and risk factors for drug-resistant-TB throughout Japan. The present study assessed drug susceptibility profiles and genetic diversity of circulating M. tuberculosis strains isolated from drug-resistant TB patients in Japan. These findings may inform development of better TB prevention and control strategies in Japan.

## RESULTS

### Epidemiology of drug-resistant TB in Japan.

A total of 15,362 culture-confirmed TB patients with known results of drug susceptibility tests (male, 63.8%; female, 36.2%) were reported from 2015 to 2016 in Japan by the Tuberculosis Surveillance Center Japan (https://www.jata.or.jp/rit/ekigaku/en/statistics-of-tb/) (TSCJ) (Table S1). Of those, 795 patients (5.2%) (male, 3.5%; female, 1.7%) were drug resistant (INH and/or RIF resistant). In the present study, we analyzed 254 culture-confirmed patients aged ≥16 years old who were diagnosed with drug-resistant TB in the period of April 2015 to March 2017 at 32 National Hospital Organization hospitals in Japan—2 in Hokkaido, 1 in Tohoku, 8 in Kanto, 4 in Chubu, 5 in Kansai, 3 in Chugoku, 2 in Shikoku, and 7 in Kyushu (Table S2 and Fig. S1). The 254 patients were approximately 32% of the 795 culture-confirmed patients reported by TSCJ as diagnosed with drug-resistant TB from 2015 to 2016 nationwide in Japan. Although it is often difficult to obtain sputum samples from children, TSCJ also reported four culture-confirmed patients aged ≥15 years old who were examined in the period 2015 to 2016, of whom drug-susceptibility profiles were unknown. In accordance with the residence regions of the 254 patients and the population sizes of those regions in Japan (Table S2), the numbers of the 254 patients analyzed per region were correlated with the regional population size (Fig. S2A and B), although the number of patients in the Tohoku region may be small relative to its population size (Fig. S2A). The numbers of drug-resistant TB patients per region were correlated with the numbers of newly diagnosed and culture-confirmed TB patients (Fig. S2C and D). These data indicate that the 254 patients represent all the drug-resistant TB patients from 2015 to 2016 in Japan. Of the 254 patients with drug-resistant TB, 174 (68.5%) were men and 80 (31.5%) were women, with a male to female ratio of 2.175 (Table 1). Of these 254 patients, 202 (79.5%) were born in Japan and 52 (20.5%) were born elsewhere. These 52 foreign-born patients were from 9 Asian countries and 1 South American country, including 18 from China (34.7%), 12 from Vietnam (23.1%), 10 from the Philippines (19.3%), and 3 from Myanmar (5.8%) (Table 2). Of the 254 patients, 194 (76.4%) were newly diagnosed and 60 (23.6%) had been previously treated for TB (Table 1). The 202 Japanese-born patients included 152 (59.8%) new and 50 (19.7%) previously treated patients, whereas the 52 foreign-born patients included 42 (16.6%) new and 10 (3.9%) previously treated patients. Of the 254 patients, 184 (72.4%) were smear-positive and 115 (45.3%) were positive for cavity formation, but there was no significant difference in the percentages of Japanese-born and foreign-born patients who were smear-positive and positive for cavity formation (data not shown).

**TABLE 1:** Patients’ gender, nationality, and history of TB^*a*^

Patient data	Japanese-born	Foreign-born	Total
Gender and birthplace			
Male	147 (57.9)^*a*^	27 (10.6)	174 (68.5)
Female	55 (21.7)	25 (9.8)	80 (31.5)
Total	202 (79.5)	52 (20.5)	254 (100)
History of TB (no. of cases)			
New	152 (59.8)	42 (16.6)	194 (76.4)
Previously treated	50 (19.7)	10 (3.9)	60 (23.6)
Total	202 (79.5)	52 (20.5)	254 (100)

a Values are numbers of patients (percentage of a total of 254 patients).

**TABLE 2:** Drug-resistant patterns and nationality of foreign-born TB patients^*a*^

Nationality	No. of foreign-born TB patients				
	Drug-resistant patterns of TB				
	INH-resistant and RIF-susceptible TB	RIF-resistant and INH-susceptible TB	MDR-TB	XDR-TB	Total (%)
China	10	2	6	0	18 (34.7)
Vietnam	9	0	3	0	12 (23.1)
Philippines	6	0	4	0	10 (19.3)
Myanmar	0	0	3	0	3 (5.8)
Indonesia	1	0	1	0	2 (3.8)
Nepal	1	0	1	0	2 (3.8)
Mongolia	0	0	2	0	2 (3.8)
India	1	0	0	0	1 (1.9)
Korea	0	1	0	0	1 (1.9)
Peru	1	0	0	0	1 (1.9)
Total	29 (55.7)	3 (5.8)	20 (38.5)	0	52 (100)

a Values are numbers of patients (%).

10.1128/mSphere.00978-20.1 FIG S1 Geographic distribution of participating hospitals (numbers of hospitals). Download FIG S1, TIF file, 0.2 MB. Copyright © 2021 Mizukoshi et al. 2021 Mizukoshi et al. https://creativecommons.org/licenses/by/4.0/ This content is distributed under the terms of the Creative Commons Attribution 4.0 International license.

10.1128/mSphere.00978-20.2 FIG S2 Geographic distribution of drug-resistant TB patients analyzed in this study (blue) and population sizes (gray) (A). (B to D) Correlation between numbers of drug-resistant TB patients in this study and the following statistical data in each region in Japan; population size (B), numbers of new TB patients published by the Tuberculosis Surveillance Center Japan in the Annual Report 2015 and 2016 (C), and numbers of culture-confirmed TB patients (D). Download FIG S2, TIF file, 0.2 MB. Copyright © 2021 Mizukoshi et al. 2021 Mizukoshi et al. https://creativecommons.org/licenses/by/4.0/ This content is distributed under the terms of the Creative Commons Attribution 4.0 International license.

10.1128/mSphere.00978-20.4 TABLE S1 Male and female patients with drug-susceptible and -resistant TB cases in 2015 to 2016 in Japan Table S1, DOCX file, 0.02 MB. Copyright © 2021 Mizukoshi et al. 2021 Mizukoshi et al. https://creativecommons.org/licenses/by/4.0/ This content is distributed under the terms of the Creative Commons Attribution 4.0 International license.

10.1128/mSphere.00978-20.5 TABLE S2 Geographical distribution of drug-resistant TB patients in this study and population size in Japan Table S2, DOCX file, 0.02 MB. Copyright © 2021 Mizukoshi et al. 2021 Mizukoshi et al. https://creativecommons.org/licenses/by/4.0/ This content is distributed under the terms of the Creative Commons Attribution 4.0 International license.

**FIG 1:**
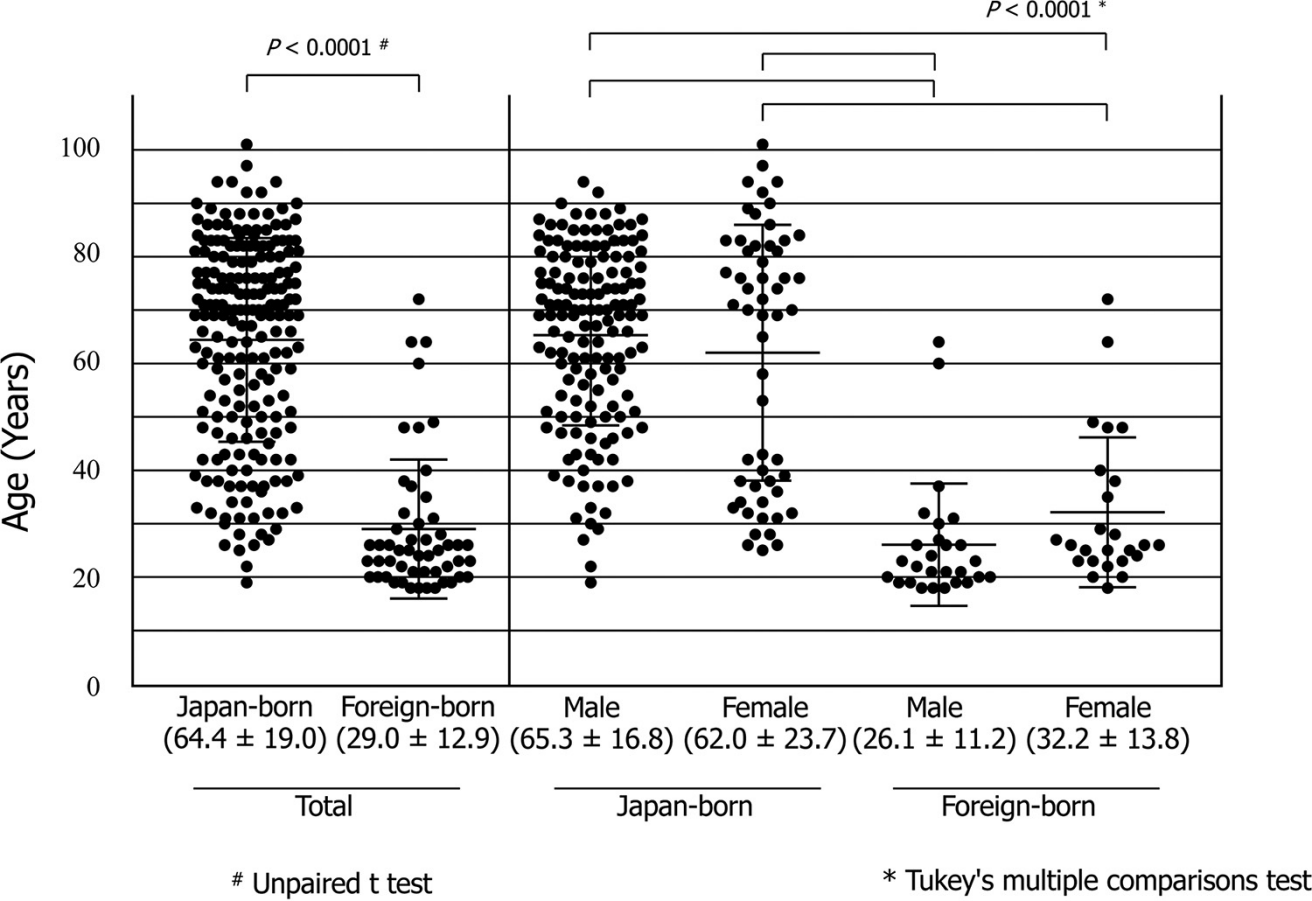
Associations among patient age, nationality, and gender. The dots indicate the age of patients at isolation of drug-resistant M. tuberculosis strains.

The average age of the 254 patients was 57.2 ± 22.9 years (range, 18 to 101 years). The foreign-born patients were significantly younger than the Japanese-born patients (29.0 ± 2.9 years versus 64.4 ± 19.0 years, *P < *0.0001 by unpaired *t* test; Fig. 1). There was no significant difference between the ages of men and women (59.2 ± 21.5 years versus 52.7 ± 25.2 years, respectively), but the ages of Japanese-born women showed a bimodal distribution (Fig. 1). As shown by histogram distribution analysis in Fig. S3, the ages of all patients tested also showed a bimodal distribution, as well as those of Japanese-born women. The first peak in the histogram of all patients was shown at age 30 and 40 years, which was due to the peaks in those of foreign-born patients aged in their 30s and Japanese-born women aged in their 40s (Fig. S3).

10.1128/mSphere.00978-20.3 FIG S3 Age distribution of TB drug-resistant TB patients in this study. Download FIG S3, TIF file, 0.6 MB. Copyright © 2021 Mizukoshi et al. 2021 Mizukoshi et al. https://creativecommons.org/licenses/by/4.0/ This content is distributed under the terms of the Creative Commons Attribution 4.0 International license.

### Drug-resistant patterns of isolates.

**FIG 2:**
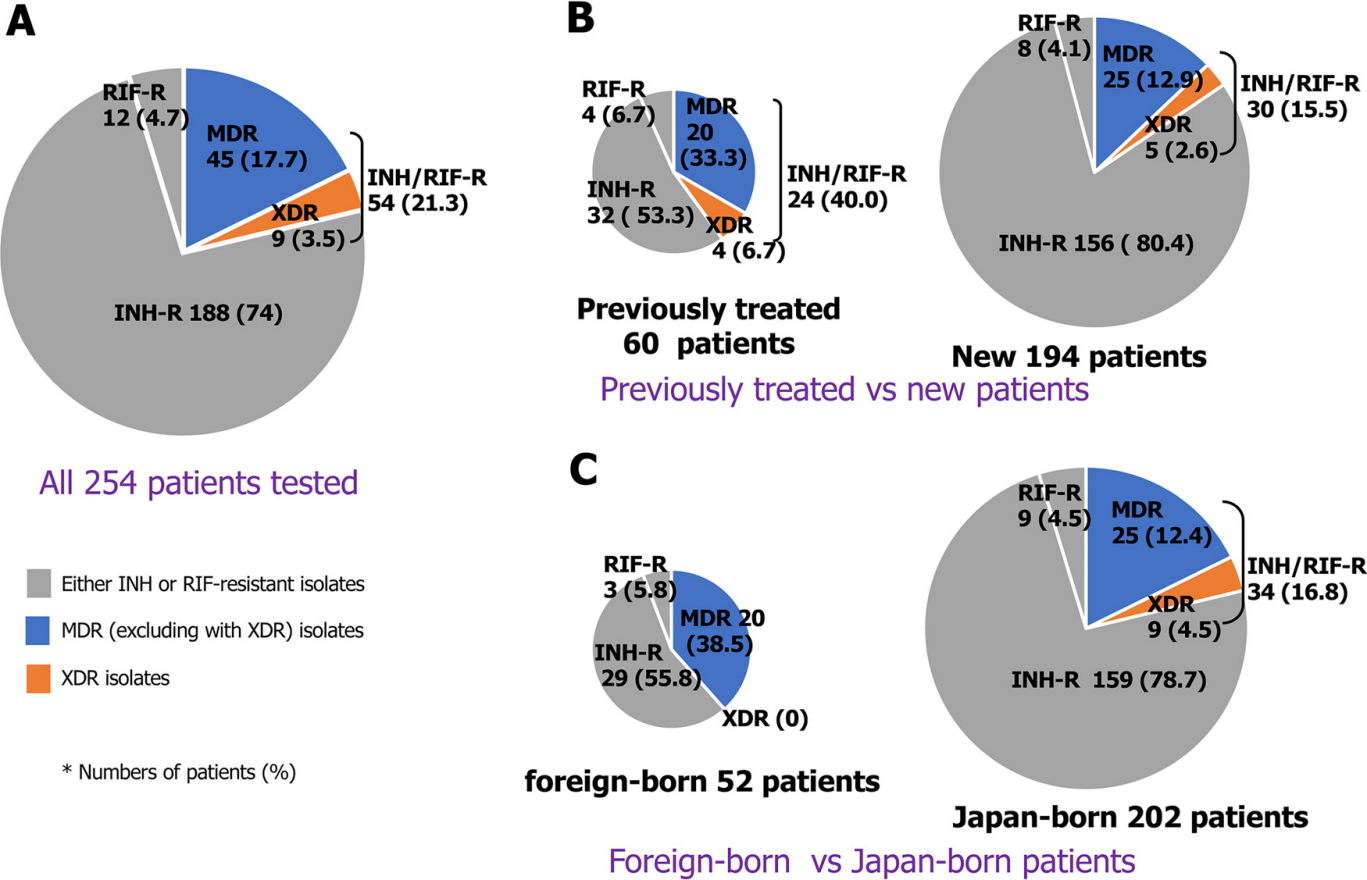
Drug-resistant patterns of TB patients. (A to C) Drug-resistant patterns of all 254 TB patients tested (A), 60 previously treated TB patients and 194 new TB patients (B), and 52 foreign-born TB patients and 202 Japan-born TB patients (C). INH-R, INH-resistant and RIF-susceptible TB; RIF-R, INH-susceptible and RIF-resistant TB; MDR, MDR TB; XDR, XDR TB.

A total of 254 M. tuberculosis isolates were obtained from 254 patients with drug-resistant TB, and the drug-resistant patterns of these isolates are shown in Fig. 2. Of these, 188 isolates (74.0%) were INH-resistant and RIF-susceptible, 12 (4.7%) were RIF-resistant and INH-susceptible, and 54 (21.3%) were resistant to both INH and RIF; these MDR isolates included 9 XDR isolates (Fig. 2A). The percentage of MDR isolates was significantly higher in previously treated than in newly diagnosed patients (Fig. 2B) (40.0% [24/60] versus 15.5% [30/194], *P = *0.0001 by Fisher’s exact test) and was significantly higher in foreign-born than in Japanese-born patients (Fig. 2C) (38.5% [20/52] versus 16.8% [34/202], *P = *0.0019 by Fisher’s exact test). All nine XDR isolates were obtained from Japanese-born patients (Fig. 2C), of mean age 68.8  ±  17.9 years, with five from patients with incipient disease, of mean age 78.2  ±  11.2 years (data not shown). Of the nine XDR isolates, four were from Kansai region (44.4%) (Table S2). Of 45 MDR excluded XDR isolates, 17 (37.8%) and 14 (31.1%) were from Kanto and Chubu, respectively (Table S2).

### Lineage analysis of drug-resistant isolates.

The lineage distribution of the 254 drug-resistant isolates is shown in Table 3. The 254 isolates were classified into four lineages as follows: 14 (5.5%) lineage 1, also known as Indo-Oceanic genotype, 199 (78.3%) lineage 2, also known as East Asian or Beijing genotype, 1 (0.4%) lineage 3, also known as East-African-Indian genotype, and 40 (15.7%) lineage 4, also known as Euro-American genotype (Table 3). Of the 199 isolates classified into lineage 2, 143 (71.9% of these, namely, 56.3% of all 254 isolates) belonged to the ancestral Beijing subgenotype, with 134 obtained from Japanese-born patients (66.3% of 202 isolates from Japanese-born patients and 93.7% of 143 ancestral Beijing subgenotype isolates). The remaining 56 isolates classified into lineage 2 belonged to the modern Beijing subgenotype, with 27 from foreign-born patients (51.9% of 52 isolates from foreign-born patients and 48.2% of the 56 modern Beijing subgenotype isolates).

**TABLE 3:** Lineage distribution of drug-resistant M. tuberculosis isolates derived from Japanese- and foreign-born patients

Lineage Beijing subgenotype	Lineage 1^*b*^	Lineage 2^*b*^			Lineage 3^*b*^	Lineage 4^*b*^	Total^*b*^
		Ancestral	Modern	Total^*a*^			
Drug-resistant isolates							
Japanese-born	8 (4.0)	134 (66.3)	29 (14.4)	163	0	31 (15.3)	202 (100)
Foreign-born	6 (11.5)	9 (17.3)	27 (51.9)	36	1 (1.9)	9 (17.3)	52 (100)
Total	14 (5.5)	143 (56.3)	56 (22.0)	199	1 (0.4)	40 (15.7)	254 (100)
INH-resistant and RIF-susceptible isolates^*c*^							
Japanese-born	8 (5.0)	108 (68.0)	18 (11.3)	126	0	25 (15.7)	159 (100)
Foreign-born	3 (10.3)	5 (17.2)	12 (41.5)	17	1 (3.4)	8 (27.6)	29 (100)
Total	11 (5.9)	113 (60.0)	30 (16.0)	143	1 (0.5)	33 (17.6)	188 (100)
RIF-resistant and INH susceptible isolates^*d*^							
Japanese-born	0	3 (33.3)	5 (55.6)	8	0	1	9 (100)
Foreign-born	0	1 (33.3)	2 (66.7)	3	0	0	3 (100)
Total	0	4 (33.3)	7 (58.3)	11	0	1 (8.3)	12 (100)
MDR isolates^*e*^							
Japanese-born	0	16 (64.0)	5 (20.0)	21	0	4 (16.0)	25 (100)
Foreign-born	3 (15.0)	3 (15.0)	13 (65.0)	16	0	1 (5.0)	20 (100)
Total	3 (6.7)	19 (42.2)	18 (40.0)	37	0	5 (11.1)	45 (100)
XDR isolates^*f*^							
Japanese-born	0	7 (77.8)	1 (11.1)	8	0	1	9 (100)
Foreign-born	0	0	0	0	0	0	0
Total	0	7 (77.8)	1 (11.1)	8	0	1	9 (100)

a Isolates belonging to lineage 2 were 80.7% (163/202), 69.2% (36/52), and 78.3% (199/254), respectively.

b Numbers of patients (%).

c Isolates belonging to lineage 2 were 79.2% (126/159), 58.6% (17/29), and 76.1% (143/188), respectively.

d Isolates belonging to lineage 2 were 88.9% (8/9), 100% (3/3), and 91.7% (11/12), respectively.

e Isolates belonging to lineage 2 were 84.0% (21/25), 80.0% (16/20), and 82.2% (37/45), respectively.

f Isolates belonging to lineage 2 were 88.9% (8/9), 0%, and 88.9% (8/9), respectively.

The 188 INH-resistant and RIF-susceptible isolates (74.0% of the 254 isolates) showed similar lineage distribution as the 254 isolates (Table 3). Most RIF-resistant and INH-susceptible isolates (11 of 12) and most MDR isolates (37 of 45) belonged to either the ancestral or modern Beijing subgenotype of lineage 2 (Table 3). Of the 19 isolates belonging to the ancestral Beijing subgenotype, 16 (84.2%) were from Japanese-born patients, whereas of the 18 isolates belonging to the modern Beijing subgenotype, 13 (72.2%) were from foreign-born patients. Of the nine XDR isolates, seven belonged to the ancestral Beijing subgenotype of lineage 2.

**FIG 3:**
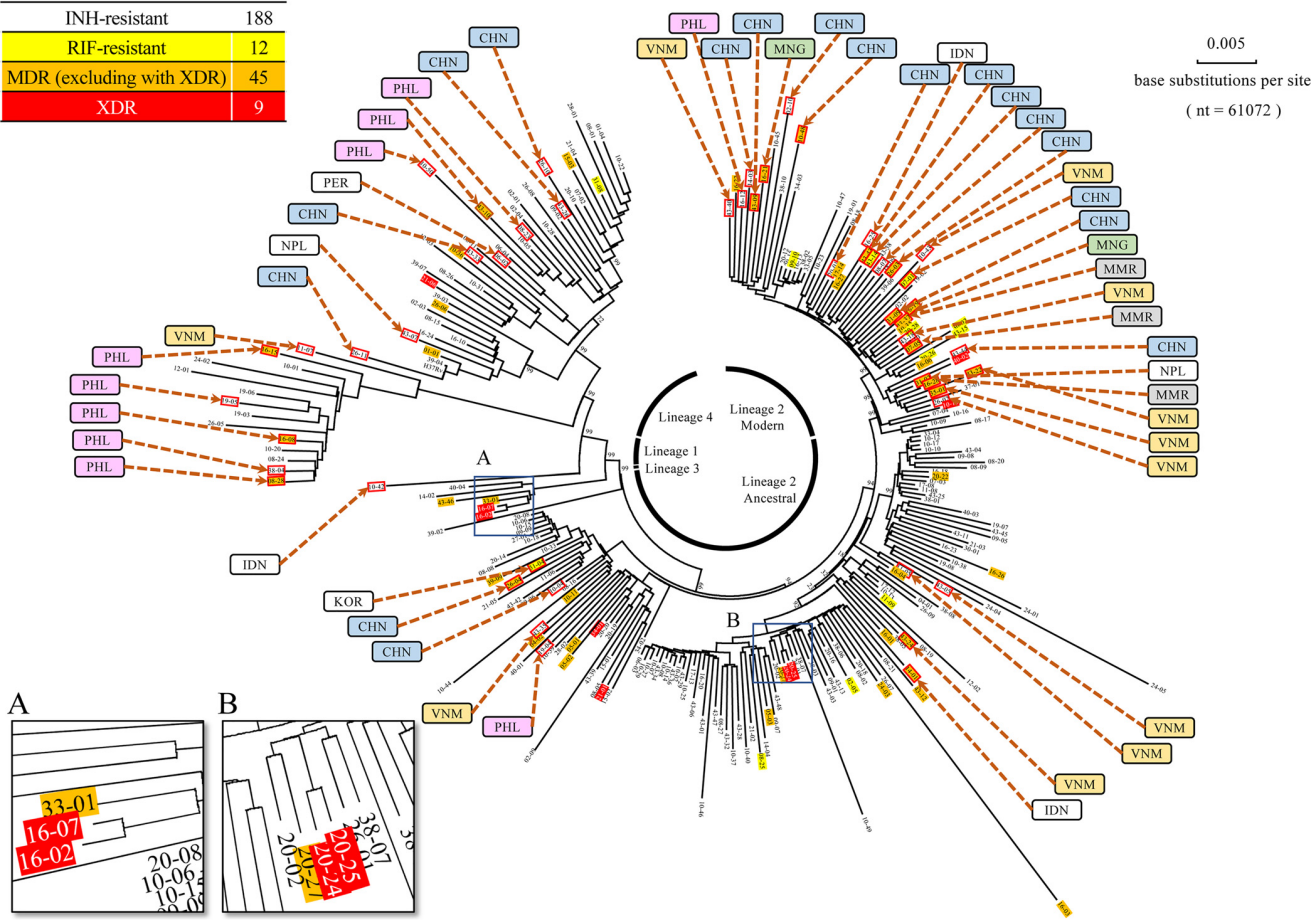
Circular phylogenetic tree of 254 drug-resistant M. tuberculosis isolates. The phylogenetic tree of the 254 clinical isolates was constructed based on SNP concatenated sequence alignments by the neighbor-joining phylogenic method. Bootstrap values are shown in the tree. Scale bars indicate nucleotide substitutions per site. Uncolored leaves indicate INH-resistant isolates; yellow, RIF-resistant isolates; orange, MDR isolates (excluding XDR isolates); red, XDR isolates. Leaves enclosed in red squares indicate isolates from foreign-born patients and the countries from where these people came (CHN, China; IDN, Indonesia; KOR, Korea; MNG, Mongolia; MMR, Myanmar; NPL, Nepal; PER, Peru; PHL, Philippines; VNM, Vietnam). The two boxes labeled A and B show that two XDR isolates were closely located.

A phylogenetic neighbor-joining (NJ) tree was constructed from these 254 drug-resistant M. tuberculosis isolates and combined with information about lineages, drug resistance patterns, and national origins (Fig. 3). Phylogenetic analysis revealed four clades, including two subclades, in good agreement with the four lineages (lineages 1 to 4) and two subtypes (the ancestral and modern Beijing subgenotypes) categorized by genotyping based on long sequence polymorphisms (LSPs). Of the 52 isolates from foreign-born TB patients, 6 (11.5%), 9 (17.3%), 27 (51.9%), 1 (1.9%), and 9 (17.3%) belonged to lineages 1, 2 (ancestral subgenotype), 2 (modern subgenotype), 3, and 4, respectively. Of the 14 isolates belonging to lineage 1, 5 were isolated from patients born in the Philippines and 1 from a patient born in Vietnam. Of the 143 isolates belonging to lineage 2 (ancestral subgenotype), 9 were from foreign-born patients—4 from Vietnam, 2 from China, and 1 each from Korea, Indonesia, and the Philippines. Of the 56 isolates belonging to lineage 2 (modern subgenotype), 27 were from foreign-born patients, including 12 from China, 7 from Vietnam, 3 from Myanmar, 2 from Mongolia, and 1 each from Nepal, Indonesia, and the Philippines. One isolate belonging to lineage 3 was from a foreign-born patient from India. Of the 40 isolates belonging to lineage 4, 9 were from foreign-born patients, including 4 from China, 3 from the Philippines, and 1 each from Nepal and Peru. As shown in Fig. 3, box A, two XDR isolates belonging to lineage 2 (ancestral subgenotype) were from the Kansai region and located close to each other, whereas another two closely related XDR isolates belonging to lineage 2 (ancestral subgenotype) were from the Chubu region and detected in different subclades (Fig. 3, box B).

## DISCUSSION

The percentage of MDR isolates was significantly higher in foreign-born TB patients (Fig. 2C) who came to Japan from Asian countries with a high TB burden, including China and South East Asian countries. The numbers of MDR-TB patients have shown annual increases in these Asian countries (8), which may reflect the higher percentage of MDR isolates in foreign-born TB patients in Japan. Of M. tuberculosis strains isolated from patients in Beijing, 9% were MDR (9). The incidence of MDR M. tuberculosis in European countries with a low TB burden is higher among migrants from countries with a high TB burden than among native-born patients (5, 6). The increased number of foreign-born TB patients, including MDR-TB patients, due to globalization has been found to affect the genetic diversity of M. tuberculosis and the distribution of lineages, not only in Tokyo (2) but also in a rural region of Japan (3). These findings emphasize the importance of monitoring M. tuberculosis isolates obtained from foreign-born patients in Japan.

Foreign-born TB patients have an impact on the nationwide genetic diversity of drug-resistant M. tuberculosis in Japan. In 2018, 1.9% of people in Japan were registered as foreign-born (12), whereas the present study showed that 20.5% of TB patients were foreign-born. Moreover, the number of foreign-born TB patients, which first exceeded 1,000 in 2012, has continued to increase (4).

Foreign-born TB patients did not have any impact on the spread of XDR M. tuberculosis in Japan, whereas Japanese-born TB patients did; i.e., all nine XDR isolates were from Japanese-born patients of mean age 68.8 years old (Fig. 2C). Five from patients with incipient TB and two pairs of XDR isolates closely related to each other were detected in the phylogenetic tree (Fig. 3, boxes A and B), indicating that small but significant numbers of XDR M. tuberculosis isolates were transmitted among the Japanese population. Although little is known about the infectivity and transmissibility of XDR M. tuberculosis, XDR M. tuberculosis isolates from patients in South Africa, a country with a high TB burden, were probably spread by person-to-person transmission rather than arising from inadequate treatment of TB (13, 14). Studies are needed to determine the epidemiology of XDR-TB spread in Japan.

The incidence of drug-resistant TB in Japanese-born women peaked at two age ranges, 30 to 39 years and 70 to 80 years old, indicating that the number of drug-resistant TB patients decreased in women aged 50 to 60 years (Fig. 1, Fig. S3). This bimodal peak was not observed in Japanese-born men, suggesting that there may be more risk factors for TB infection in Japanese men than in Japanese women.

The present study found that men were more susceptible to drug-resistant TB than women, with a male to female ratio in Japan of 2.175:1. This ratio was markedly higher than the ratio of male to female TB patients in Japan of 1.475:1 (1), indicating that the high male to female ratio of drug-resistant TB patients was not due only to the high male to female ratio of TB patients. These results are consistent with a report in China that the ratio of drug-resistant TB patients was higher in males than in females from 2004 to 2019 in Shandong, China (15), whereas the ratios of drug-resistant TB patients were higher in females than in males in 2013 to 2018 in Canada (16). Other factors may therefore contribute to differences in these ratios, such as social/medical behavior and susceptibility to drug-resistant TB.

There are few studies of MDR M. tuberculosis based on whole-genome sequencing at the nationwide scale in Asian countries (10, 11). MDR M. tuberculosis belonging to lineage 2 caused an ongoing MDR M. tuberculosis epidemic throughout Central Asia (10). Of drug-resistant M. tuberculosis isolates, 83.2% belonged to lineage 2 in Thailand, and clusters of these isolates contributed to the high prevalence of drug-resistant TB in that country (11). Those results are consistent with the findings of the present study. Whole-genome sequencing analysis will become one of the essential tools to investigate the epidemiology of drug-resistant tuberculosis.

## MATERIALS AND METHODS

### Clinical isolates.

A total of 254 clinical isolates of M. tuberculosis resistant to INH and/or RIF were obtained from 254 patients who had been diagnosed with drug-resistant TB from April 2015 to March 2017 at 32 National Hospital Organization hospitals throughout Japan; written informed consent was obtained from each participant or from the parents of minors, and their clinical information was anonymized.

### Drug susceptibility testing.

Clinical isolates were analyzed using an agar proportion method with Wellpack (Japan BCG Laboratory, Tokyo, Japan), which is based on a slightly modified WHO protocol (17). In the modified protocol, egg-based Ogawa medium containing 2,3-diphenyl-5-thienyl-(2)-tetrazolium chloride (STC) at 50 μg/ml was plated into 16-well plates. STC resulted in reliable and more easily interpretable data. A 10-μl loop of a colony of the isolates grown on Ogawa medium was inoculated into 4.5 ml of 7H9 medium and incubated for 5 to 7 days. The bacterial suspension in 7H9 was adjusted by adding sterile water with a density equivalent to that of a 1 McFarland turbidity standard. Separate 10^−2^ and 10^−4^ dilutions of the adjusted bacterial suspension were prepared, and 50 μl of the 10^−2^ dilution was inoculated onto a control well and drug-containing wells which contained INH (0.2 μg/ml and 1.0 μg/ml), rifampicin (RIF) (40 μg/ml), ethambutol (EB) (2.5 μg/ml), kanamycin (KM) (20 μg/ml), *p*-aminosalicylic acid (PAS) (0.5 μg/ml), streptomycin (SM) (10 μg/ml), ethionamide (TH) (20 μg/ml), enviomycin (EVM) (20 μg/ml), cycloserine (CS) (30 μg/ml), and levofloxacin (LVFX) (1.0 μg/ml). Similarly, 50 μl of the 10^−4^ dilution was inoculated into another control well. The 16-well plates were sealed, to prevent the wells from drying by evaporation, and incubated at 37°C. The plates were examined carefully each week for a period of no longer than 3 weeks.

### DNA preparation and whole-genome sequencing.

Genomic DNA of M. tuberculosis clinical isolates was prepared as described previously (18). Briefly, M. tuberculosis isolates were grown on egg-based Ogawa medium for 3 to 4 weeks. All bacterial cells from one slant were transferred to 400 μl of TE buffer (10 mM Tris-HCl, 1 mM EDTA [pH 8.0]), and the solution was heated at 80°C for 20 min to kill bacteria. Then, 50 μl of lysozyme (10 mg/ml) was added, and the tube was incubated overnight at 37°C. Next, 70 μl of sodium dodecyl sulfate (10%) and 5 μl of proteinase K (10 mg/ml) were added, and the mixture was incubated for 10 min at 65°C. A 100-μl volume of 5 M NaCl and the same volume of an *N*-cetyl-*N*,*N*,*N*-trimethylammonium bromide (CTAB)-NaCl solution (4.1 g of NaCl and 10 g of CTAB per 100 ml) were added together. The tubes were vortexed and incubated for 10 min at 65°C. An equal volume of chloroform-isoamyl alcohol (24:1) was added, the mixture was centrifuged for 5 min at 12,000 × *g*, and the aqueous supernatant was carefully transferred to a fresh tube. The total DNA was precipitated in isopropanol and was redissolved in 20 μl of TE buffer. Genomic DNA eluates were quantified using the Qubit double-stranded DNA (dsDNA) BR assay kit (Thermo Fisher Scientific, Waltham, MA). A DNA library was prepared from each extracted DNA sample using a Nextera XT DNA library prep kit. The DNA library was quantified using a high-sensitivity DNA kit and an Agilent 2100 bioanalyzer (Santa Clara, CA). Paired-end multiplexed Illumina sequencing was performed using a MiSeq system (Illumina, Inc., San Diego, CA). Briefly, a Nextera XT DNA library was prepared from each extracted DNA sample. Each DNA library was sequenced on the MiSeq system (Illumina) to obtain short reads with 300-bp paired-end reads. The MiSeq run was performed using a Nextera XT index kit setA and MiSeq reagent kit v3. The raw reads with a Q30 score of >74.0% were used for the whole-genome sequences of the clinical isolates.

### CASTB analysis.

Whole-genome sequencing (WGS) data, generated with next-generation sequencers, were analyzed using the comprehensive analysis server for M. tuberculosis complex (CASTB) (http://castb.ri.ncgm.go.jp/CASTB/) (19). The results of virtual lineage analysis based on long sequence polymorphisms (LSP) and single nucleotide polymorphism (SNP) concatemers were automatically obtained from sequence data. Phylogenetic analysis was performed using the neighbor-joining (NJ) method. SNPs identified by CASTB were concatenated, and the NJ phylogenetic tree was generated by aligning concatenated SNPs with MEGA v7 (20).

### Statistical analyses.

Data were summarized as mean, median, and/or range, as appropriate, and compared using Fisher’s exact tests or nonparametric Student’s *t* tests. All tests were two-tailed, with *P  <  *0.05 considered statistically significant. All statistical analyses were performed using Prism v8 (GraphPad Software, San Diego, CA, USA).

### Ethics approval and consent to participate.

The study protocol was carefully reviewed and approved by the National Hospital Organization Central Research Ethics Committee (H30-0205009) and by the ethics committee of the National Center for Global Health and Medicine (NCGM-G-002058-01).
