# Supplementary figures and images for: Molecular Epidemiology of Drug-Resistant Mycobacterium Tuberculosis in Japan
Source: mSphere. 2021 Jul 7;6(4):e00978-20. doi: 10.1128/mSphere.00978-20 (PMC8386464; doi:10.1128/mSphere.00978-20)

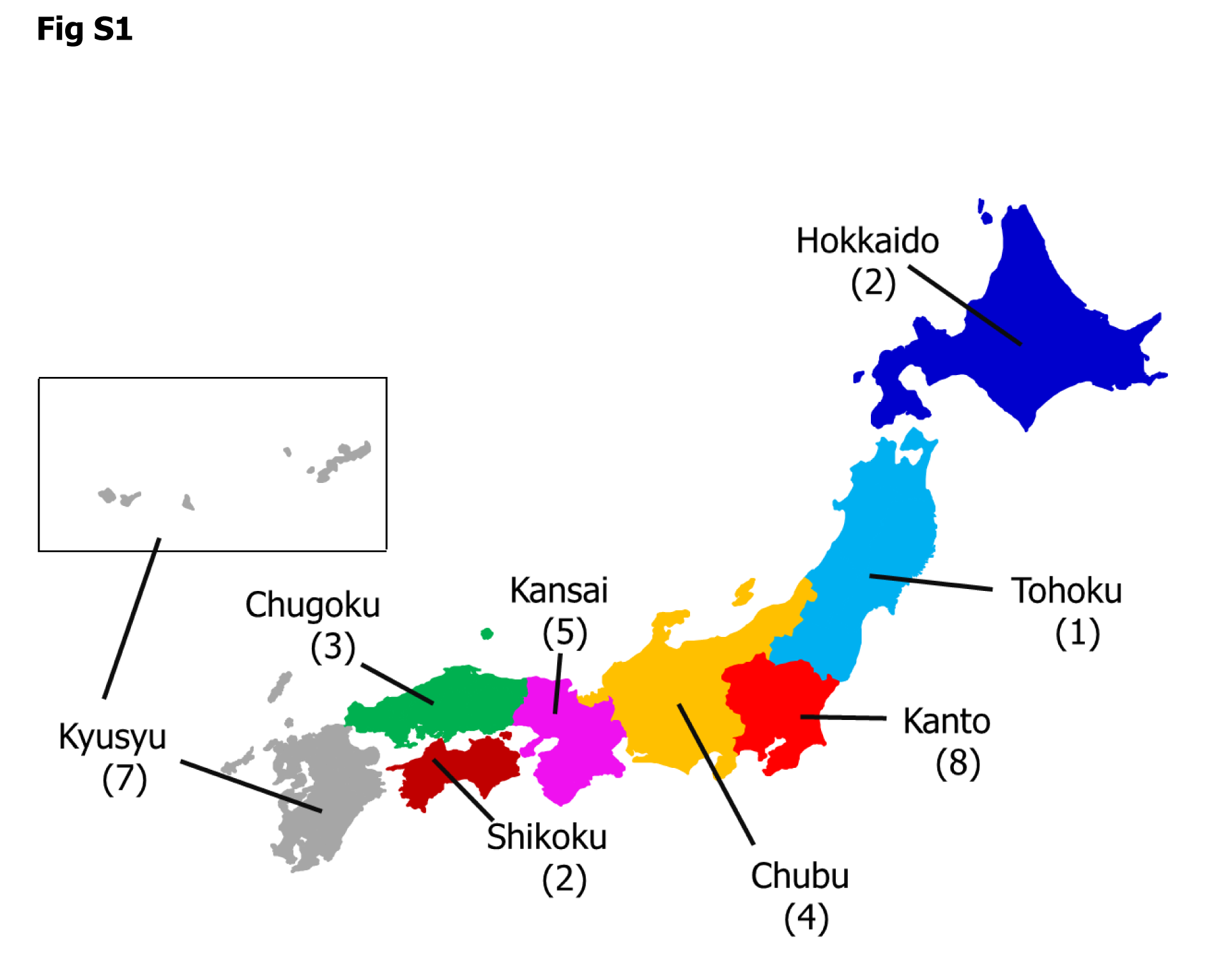

Supplement: FIG S1 [file msphere.00978-20-sf001.tif]

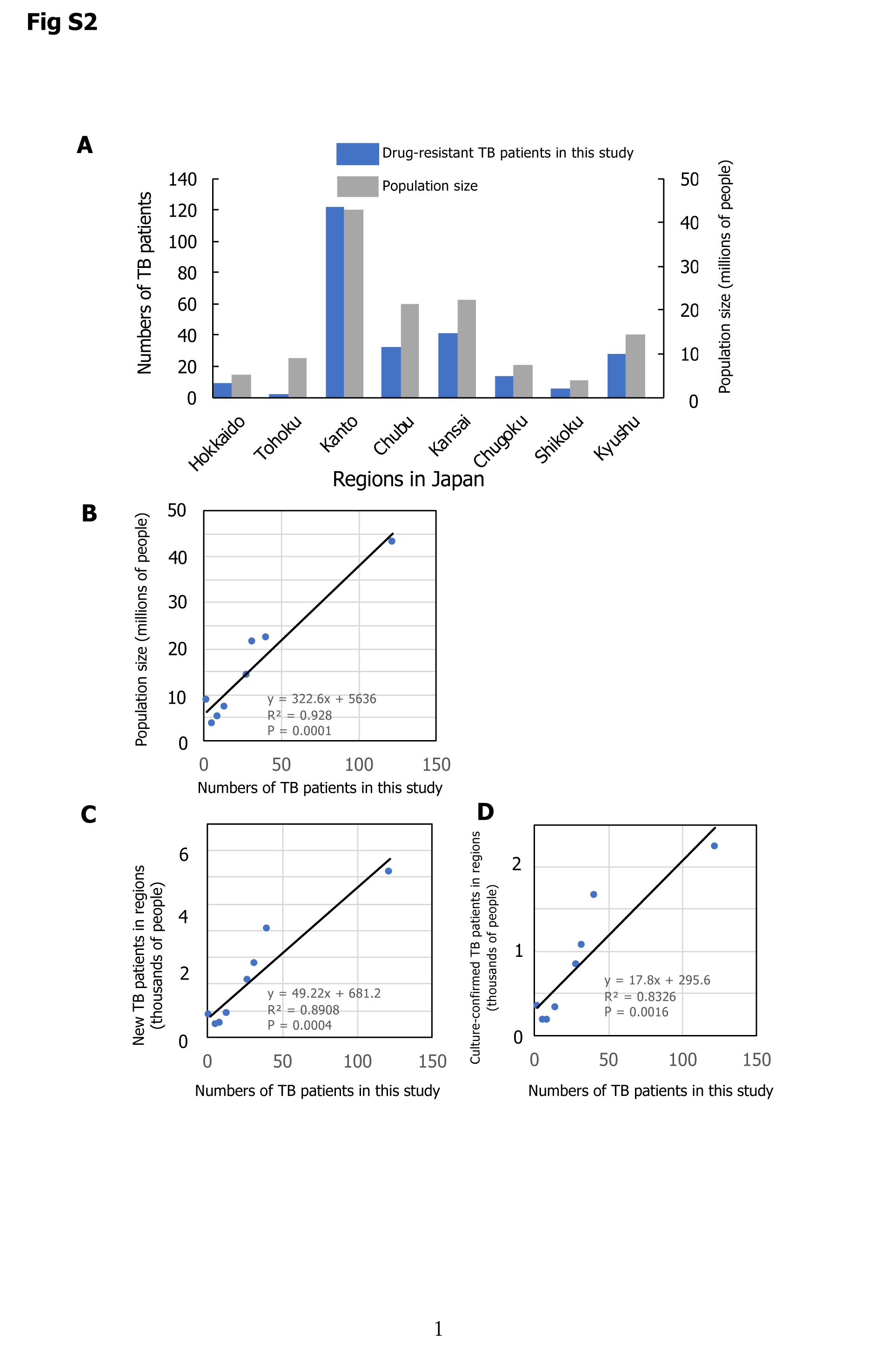

Supplement: FIG S2 [file msphere.00978-20-sf002.tif]

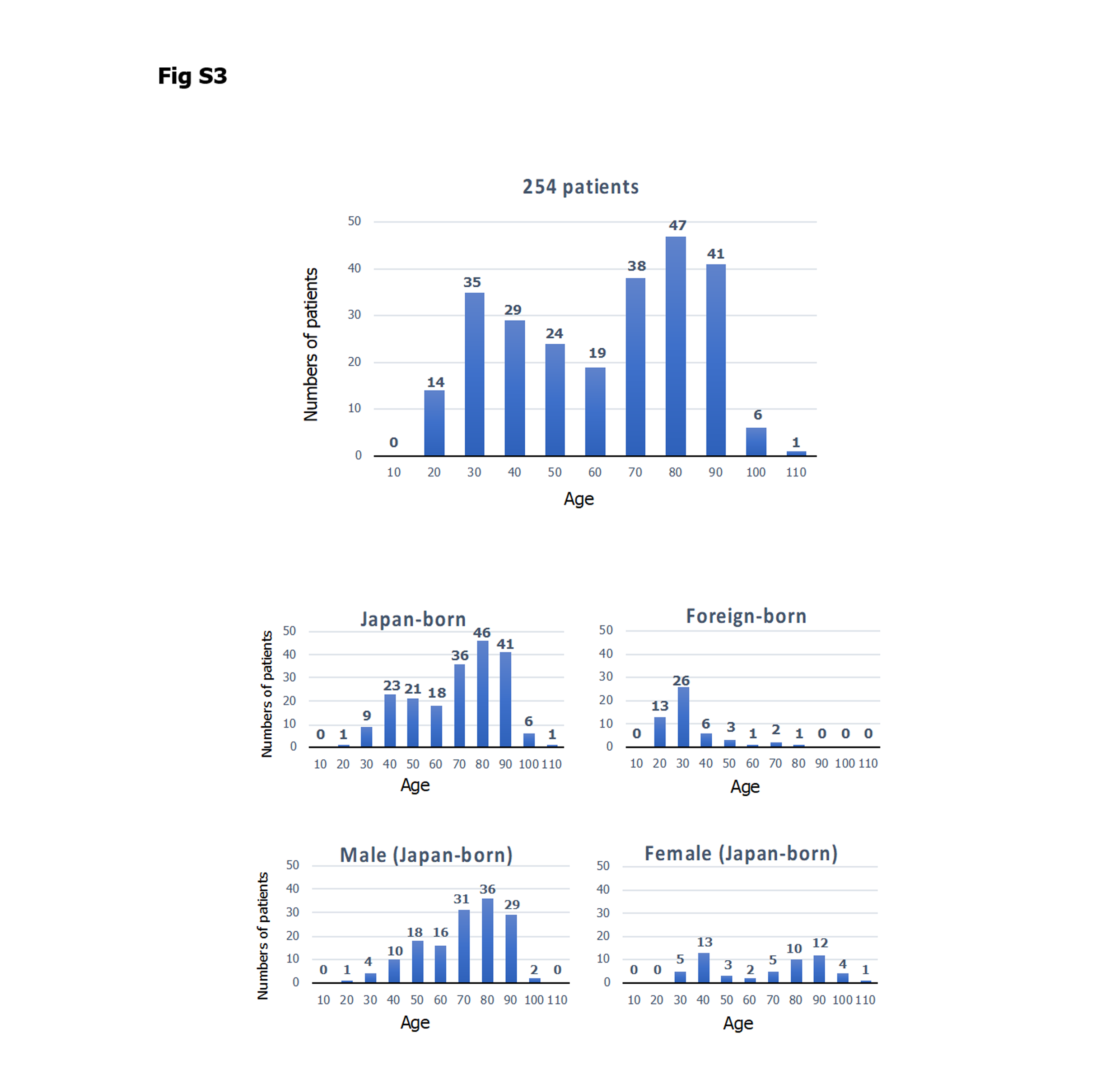

Supplement: FIG S3 [file msphere.00978-20-sf003.tif]
