# Supplementary material for: Molecular Epidemiology of Drug-Resistant Mycobacterium Tuberculosis in Japan
Source: mSphere. 2021 Jul 7;6(4):e00978-20. doi: 10.1128/mSphere.00978-20 (PMC8386464; doi:10.1128/mSphere.00978-20)
Supplement: TABLE S1 [file msphere.00978-20-st001.docx]

**TABLE S1** Male and female patients with drug-susceptible and -resistant TB cases in 2015-2016 in Japan^a^

|  | Numbers of culture-confirmed TB cases with | | | |  |
| --- | --- | --- | --- | --- | --- |
| Gender of patients | known drug susceptibility test results for INH/RFP | MDR isolates | INH-resistant and RFP-susceptible isolates | INH-susceptible and RFP-resistant isolates | Total of INH- and/or RIF- resistant isolates |
| Male | 9,804 (63.8) | 59 (0.4) | 442 (2.9) | 32 (0.2) | 533（3.5） |
| Female | 5,558 (36.2) | 38 (0.2) | 202 (1.3) | 22 (0.1) | 262（1.7） |
| Total | 15,362 (100) | 97 (0.6) | 644 (4.1) | 54 (0.3) | 795（5.2） |

^a^ Data were calculated from Annual Reports 2015 and 2016 published by the Tuberculosis Surveillance Center Japan (<http://www.jata.or.jp/rit/ekigaku/en>

^b^ Values are numbers of patients (% of a numbers of patients)
