# Supplementary material for: Molecular Epidemiology of Drug-Resistant Mycobacterium Tuberculosis in Japan
Source: mSphere. 2021 Jul 7;6(4):e00978-20. doi: 10.1128/mSphere.00978-20 (PMC8386464; doi:10.1128/mSphere.00978-20)
Supplement: TABLE S2 [file msphere.00978-20-st002.docx]

**TABLE S2** Geographic distribution of drug-resistant TB patients in this study and population size in Japan

| Region | Numbers of  hospitals |  | Numbers of drug-resistant TB patients | | | Population size,  thousands of people^a^  (% of a total population) |
| --- | --- | --- | --- | --- | --- | --- |
|  |  |  |  | MDR | XDR |  |
| Hokkaido | 2 |  | 9 | 1 | 0 | 5367 (4.2) |
| Tohoku | 1 |  | 2 | 1 | 0 | 8949 (7.0) |
| Kanto | 8 |  | 122 | 17 | 1 | 43063.5 (33.9) |
| Chubu | 4 |  | 32 | 14 | 2 | 21437.5 (16.9) |
| Kansai | 5 |  | 41 | 4 | 4 | 22515 (17.7) |
| Chugoku | 3 |  | 14 | 3 | 0 | 7422 (5.8) |
| Shikoku | 2 |  | 6 | 2 | 0 | 3831.5 (3.0) |
| Kyushu | 7 |  | 28 | 3 | 2 | 14427.5 (11.4) |
| Total | 32 |  | 254 | 45 | 9 | 127014 (100.0) |

^a^Population size of regions show the average for 2015 and 2016 in Japan.
